# Trial Participants’ Perceptions of the Impact of Ecological Momentary Assessment on Smoking Behaviors: Qualitative Analysis

**DOI:** 10.2196/52122

**Published:** 2024-01-16

**Authors:** Elizabeth R Stevens, Rina Li, Grace Xiang, Rachel Wisniewski, Sidney Rojas, Katherine O'Connor, Olivia Wilker, Mahathi Vojjala, Omar El-Shahawy, Scott E Sherman

**Affiliations:** 1 Department of Population Health, NYU Grossman School of Medicine, New York, NY, United States; 2 School of Global Public Health, New York University, New York, NY, United States

**Keywords:** smoking behavior, ecological momentary assessment, bias, behavioral impact, smoking, smoker, qualitative analysis, pilot study, tool, data collection, tobacco, text message, accountability, mHealth, mobile health, message, trigger, cigarette

## Abstract

**Background:**

Ecological momentary assessment (EMA) is an increasingly used tool for data collection in behavioral research, including smoking cessation studies. As previous addiction research suggests, EMA has the potential to elicit cue reactivity by triggering craving and increasing behavioral awareness. However, there has been limited evaluation of its potential influence on behavior.

**Objective:**

By examining the perspectives of research participants enrolled in a tobacco treatment intervention trial, this qualitative analysis aims to understand the potential impact that EMA use may have had on smoking behaviors that may not have otherwise been captured through other study measures.

**Methods:**

We performed a qualitative analysis of in-depth interviews with participants enrolled in a pilot randomized controlled trial of a tobacco treatment intervention that used SMS text messaging to collect EMA data on smoking behaviors. In the pilot randomized controlled trial, combustible cigarette and e-cigarette use and smoking-related cravings were measured as part of an EMA protocol, in which SMS text messaging served as a smoking diary. SMS text messaging was intended for data collection only and not designed to serve as part of the intervention. After a baseline assessment, participants were asked to record daily nicotine use for 12 weeks by responding to text message prompts that they received 4 times per day. Participants were prompted to share their experiences with the EMA text messaging component of the trial but were not directly asked about the influence of EMA on their behaviors. Transcripts were coded according to the principles of the framework for applied research. The codes were then examined, summarized, and grouped into themes based on the principles of grounded theory.

**Results:**

Interviews were analyzed for 26 participants. The themes developed from the analysis suggested the potential for EMA, in the form of an SMS text messaging smoking diary, to influence participants’ smoking behaviors. The perceived impacts of EMA text messaging on smoking behaviors were polarized; some participants emphasized the positive impacts of text messages on their efforts to reduce smoking, while others stressed the ways that text messaging negatively impacted their smoking reduction efforts. These contrasting experiences were captured by themes reflecting the positive impacts on smoking behaviors, including increased awareness of smoking behaviors and a sense of accountability, and the negative impacts on emotions and smoking behaviors, including provoking a sense of guilt and triggering smoking behaviors.

**Conclusions:**

The collection of EMA smoking behavior data via SMS text messaging may influence the behaviors and perceptions of participants in tobacco treatment interventions. More research is needed to determine the magnitude of impact and mechanisms, to account for the potential effects of EMA. A broader discussion of the unintended effects introduced by EMA use is warranted among the research community.

## Introduction

Ecological momentary assessment (EMA) is a data collection method that is increasingly being used in health and behavioral sciences [1,2]. EMA has been shown to be a useful tool for measuring behaviors associated with substance use [3]. Consequently, there has been a strong interest in the use of EMA in smoking cessation studies [4]. However, as indicated in previous addiction research, EMA has the potential to elicit cue reactivity by triggering craving and increasing behavioral awareness [5-8]. As such, there is potential for the use of EMA to create an assessment effect and inadvertently influence behaviors in some settings.

*Assessment effects* refer to the phenomenon in which the outcome of interest (eg, a behavior) is modified simply by assessing it, arising due to the assessment method or its interaction with the intervention [9,10]. The frequent prompts for self-reporting that are often required by EMA might inadvertently influence participants’ behaviors by, among other mechanisms, increasing their self-awareness, altering an emotional response, or serving as reminders of the behavior [2,8,11,12]. Depending on the behavior of interest, these EMA consequences may differentially impact outcomes. These assessment effects can have a significant impact on the interpretation of trial results but are rarely considered in trial design [13].

Despite its potential importance to the interpretation of research results, there are a limited number of studies investigating the potential for EMA to produce an assessment effect. Within the research that does exist, there have been mixed results reported, with some studies reporting no impact on behavior [11,14,15] and others indicating that EMA likely has an impact [2,5-8,11,12,14]. For this reason, it is important to understand if and how the use of EMA data collection in smoking research could impact smoking behaviors.

To better understand the potential impact of using EMA to measure smoking behaviors, we performed a qualitative analysis of in-depth interviews with participants in a randomized controlled trial (RCT) pilot study of a smoking intervention that used SMS text messaging–based EMA as a data collection strategy [16]. By examining the perspectives of the trial participants, this qualitative analysis aims to understand the potential impact that the use of EMA may have had on smoking behaviors that may not have otherwise been captured through other means of data collection.

## Methods

### Study Design

For the purposes of assessing the acceptability of a smoking intervention and determining points for potential program improvement, we performed a qualitative analysis of in-depth interviews conducted with participants of an RCT pilot study that compared the effectiveness of behavioral counseling and the use of e-cigarettes on smoking outcomes to that of behavioral counseling and nicotine replacement therapy (NRT).

### Ethical Considerations

The interviews and analysis procedures were reported in accordance with the COREQ (Consolidated Criteria for Reporting Qualitative Research) checklist (Checklist 1), as applicable [17]. The study protocol was approved by the New York University Langone Health Institutional Review Board (approval number i20-00839), and written documentation of informed consent was received prior to starting data collection. Participants were provided with a US $20 incentive for their participation. Participant data were maintained on a secure server. After transcription, all participant data were deidentified prior to data analysis.

### Setting and Participants

Interview participants were recruited upon completion of the intervention phase of the RCT at the 12-week follow-up study visit. All RCT participants were invited to participate in an in-depth interview to discuss their experiences with the intervention and other aspects of the trial. Interview recruitment ended once thematic saturation was reached. Interviews lasted approximately 30 minutes and were performed between April 2021 and November 2022.

The pilot RCT was performed to determine the feasibility and acceptability of an e-cigarette–based smoking intervention and to compare the effectiveness of counseling and e-cigarette use on smoking outcomes to that of counseling and NRT [16]. Text message–based EMA data collection was used to record smoking patterns. Patients from the electronic health record system of New York University Langone Health—a private hospital system serving New York, New Jersey, and Connecticut—were recruited as RCT participants. The RCT participant sample was initially restricted to patients with a diagnosis of chronic obstructive pulmonary disease, but the scope was later expanded to include patients with a diagnosis of coronary artery disease, peripheral artery disease, or asthma. In addition, to be eligible, RCT participants were required to smoke ≥4 days per week, with at least 5 cigarettes smoked on the days that participants did smoke; be motivated to quit smoking; and possess a phone with SMS text messaging capabilities. A total of 121 participants were recruited into the pilot RCT.

### RCT EMA Protocol

**Figure 1.**
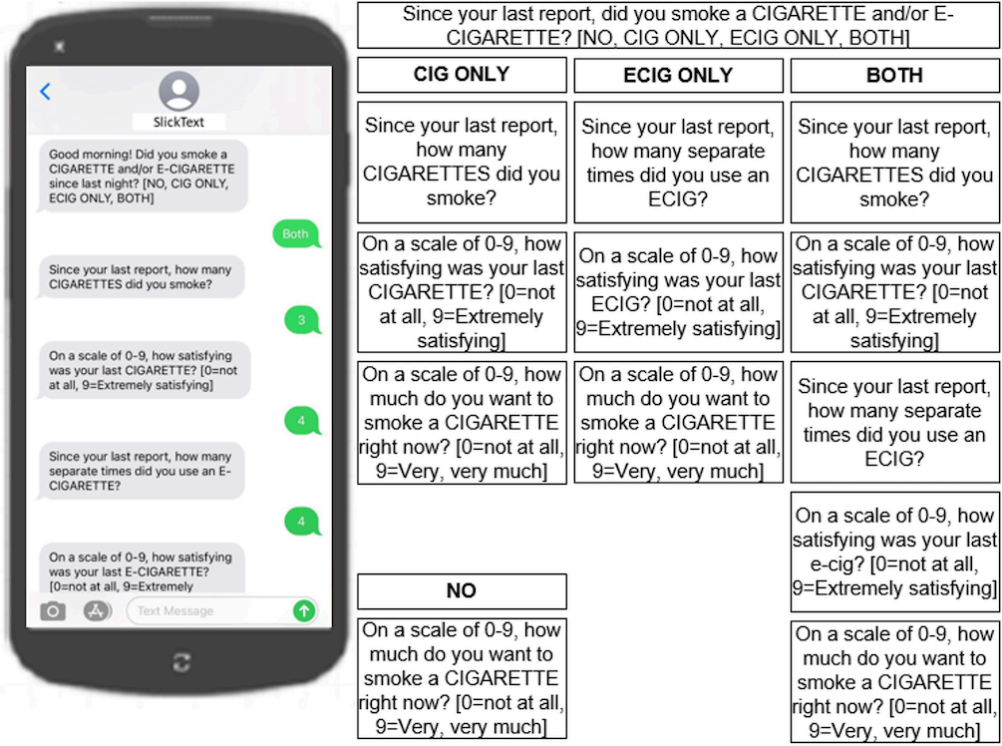
Sample of ecological momentary assessment texting prompts. CIG: cigarette; ECIG: e-cigarette.

In the pilot RCT, combustible cigarette use and e-cigarette use, as well as smoking-related cravings, were measured as part of an EMA protocol, in which SMS text messaging served as a smoking diary. The SMS text messaging was intended for data collection only and not designed to serve as part of the intervention. After a baseline assessment, participants were asked to record their daily nicotine use for 12 weeks by responding to text message prompts that they received 4 times per day. The coverage design prompted participants to provide brief check-in reports via text message over the course of each day, wherein they were asked to report combustible cigarette and e-cigarette use based on their study arm (Figure 1). Responses to 1-item measures of cigarette craving and satisfaction were also collected from each report.

### Data Collection

Semistructured, in-depth telephone interviews, which lasted approximately 30 minutes, were conducted by research staff (OW and MV). Interviews were audio-recorded, transcribed, and imported into Dedoose software (SocioCultural Research Consultants LLC) for qualitative data analyses.

The interview guide covered topics that were designed for the following goal: gaining a deeper understanding of the participants’ experiences, intervention satisfaction, attitudes toward e-cigarettes, and intentions to quit. The interviews were intended to assist with the further adaptation of the e-cigarette smoking intervention and behavioral counseling manual used in the RCT. The interview guide was developed based on the pilot RCT procedures to provide additional insights about the barriers and facilitators of e-cigarette use and how to refine the current approach to enhance program retention and outcomes. Interviews covered topics such as program aspects that the participants liked or disliked, features of the intervention that should be modified, participants’ experiences with using e-cigarettes or NRT, intentions of using e-cigarettes after the intervention, and whether participants’ health symptoms interfered with their ability to engage in the intervention. The participants were prompted to share their opinions on the EMA texting component of the trial but were not directly asked about the influence of the EMA on their behaviors. Participants were prompted to discuss the texting in the interview, as follows: “During the program, you answered questions over text on daily basis—What was that experience like for you?” This was followed by probes, including “How may texting have affected your overall experience with the program?” A copy of the interview script is available in Multimedia Appendix 1.

### Data Analysis

Interview transcripts were coded by using procedures that were designed to ensure thoroughness and reliability. We used Dedoose software to manage the data and coded the data according to the principles of the framework for applied research [18], which consists of the following 5-stage process: familiarization, identifying themes, indexing, charting, and interpretation. Codes were primarily developed a priori based on intervention components and the quality improvement goals of this study. Additional codes were developed by reviewing a random sample of interviews and via discussion with the coding team. The general development of themes arose from the data, using the principles of grounded theory [19]. To enhance reliability, 2 researchers took part in the coding and analysis process for each interview. Prior to full coding, a random sample of interviews was double coded, and intercoder reliability was assessed based on percent agreement (range 79.5%-87.9%). Afterward, all disagreements were discussed as a group to improve concordance among coders. All interviews were then independently coded by 5 coders (GX, KO, RL, RW, and SR) who worked in various pair combinations; each pair met with the other coders and a coauthor (ERS) to resolve discrepancies. When coding was completed, the quotations for each code were examined, summarized, and grouped together into themes.

## Results

### Participants

Interviews were performed with a total of 27 participants. Due to an audio malfunction, 1 interview was not included in the analysis. As such, 14 participants were in the e-cigarette study arm and 12 were in the NRT study arm. The average age of the participants included in the analysis was 57.1 (range 28-74) years; 54% (14/26) of participants identified as female, 46% (12/26) were White, 31% (8/26) were Black, 15% (4/26) were Hispanic, and 4% (1/26) were Asian. The majority (17/26, 65%) of participants had completed at least some college; 35% had a high school education or less. Around two-thirds (17/26, 65%) of participants had a diagnosis of chronic obstructive pulmonary disease, 15% (4/26) of participants were diagnosed with coronary artery disease or peripheral artery disease, and 19% (5/26) of participants were diagnosed with asthma.

### Themes

#### Overview of Themes

Without being directly prompted, 18 of the 26 participants described EMA impacting their behaviors or emotions, and several themes indicating a potential, inadvertent influence of EMA on smoking behaviors emerged. The perceived impacts of EMA texting on smoking behaviors were polarized; some participants emphasized the positive impacts of the text messages on their efforts to reduce smoking, while others stressed the ways in which the texting negatively impacted their smoking reduction efforts. These contrasting experiences were captured by 2 major themes and their subthemes, reflecting the positive impacts on smoking behaviors, including increased awareness of smoking behaviors and a sense of accountability, and the negative impacts on emotions and smoking behaviors, including provoking a sense of guilt and triggering smoking behaviors (Table 1).

**Table 1.** Major themes and subthemes.

Themes and subthemes			Frequency, n
**EMA^a^ texting has a positive impact on smoking behaviors**			16
	**EMA texting serves as a source of accountability**		13
		Anticipation of the next text message serves as a deterrent to impulsive smoking	6
	Texting prompts increase awareness of smoking habits		16
	**Reminders of the goal to reduce cigarette smoking**		11
		Check-ins serve as markers of progress made toward quitting	5
**Negative impact of EMA texting on emotions and smoking behaviors**			7
	Repeated text messages asking about smoking behaviors produce negative emotions		4
	Text messages inquiring about cigarette use may have a triggering effect		5

a EMA: ecological momentary assessment.

#### Perceived Positive Impacts of EMA Texting Procedures

##### Overview of Positive Impacts

A major theme that arose was the perceived positive impacts of the EMA methods on efforts to reduce smoking, as many (n=16) participants perceived the SMS text messaging smoking diary as an important component of the tobacco treatment intervention and their experience during their efforts to reduce their cigarette smoking. Participants described the EMA text messages as helpful because “you could use [them] for yourself as a tool” (Participant E09), and while stating that “the text messages are a pain in the butt,” a participant thought that “they were very useful.…A useful pain in the butt” (Participant E04). Within this overarching theme—the positive influence of EMA—four subthemes that highlighted the potential roles of EMA text messages emerged: EMA text messages (1) increase awareness of smoking habits, (2) serve as reminders of smoking goals, (3) mark progress made, and (4) provide a sense of accountability.

##### Texting Prompts Increase Participants’ Awareness of Their Smoking Habits

Many (n=16) participants noted that the EMA text messages helped them with their smoking behaviors because the act of recording the number of cigarettes smoked increased their awareness of their smoking behaviors. This theme—increased smoking habit awareness—encompassed the following three layers: (1) awareness of the number of cigarettes smoked and smoking habits, (2) reminders of the goal to reduce cigarette smoking, and (3) markers of progress made.

The predictable and repeated EMA text message requests for participants to report the number of cigarettes smoked prompted reflection on their smoking habits. By engaging in the conscious effort of quantifying the number of cigarettes smoked, the participants heightened their self-awareness and gained a clearer understanding of the frequency of their cigarette use. One participant remarked:

[The text message would say] “…have you smoked? When was the last time you smoked your last cigarette?” So, it helped me to be aware of how many cigarettes I was smoking per day.

[Participant N24]

With regard to the act of recording cigarette use, a participant noted:

…you’re so aware with the…text messages…of just how often you smoke.…Because with the text, I was physically writing it and seeing it.

[Participant E27]

##### Reminders of the Goal to Reduce Smoking

For some participants (n=11), the EMA text messages also served as reminders of their goal to reduce or quit smoking and heightened their sense of purpose and determination. One participant said, “It [the text messaging] does remind you what you’re supposed to be focused on” (Participant N20), and in this way, the messages served as frequent reminders of participants’ intentions to change their smoking behaviors. The messages also invigorated their commitment to achieving these goals; one participant said:

It [the text messages] kept me going. It kept me wanting to quit, you know, and to keep doing it, to keep at the program.

[Participant N36]

The text messages also kept participants feeling involved:

…[questions] like, “how many cigarettes have you smoked?”…kind of keep you involved in it instead of letting it go on the way side. Like it would kind of keep reminding you that…this is what you’re working on, you know?

[Participant N50]

##### Markers of Progress Made Toward Smoking Reduction

The EMA text messages served as markers of progress made toward reducing smoking (n=5). By regularly quantifying and reporting their smoking behaviors, participants saw tangible evidence and took note of the accomplishments resulting from their efforts. By tracking progress over time, participants observed patterns of improvement, which reinforced their motivation to continue cutting back on smoking. When describing why they found the text messages useful, one participant said:

…it’s like a progress type thing. So, I enjoyed the texts.…It kind of gave me a reminder [of my progress] because as I went on, I had less and less craving.

[Participant N58]

##### EMA Texting Serves as a Source of Accountability for Progress Toward Reducing Cigarette Smoking

Many participants (n=13) believed that the EMA text messages had a positive effect on their sense of accountability in their efforts to reduce smoking. Knowing that they would receive inquiries about their smoking habits increased participants’ mindfulness in reducing their smoking. One participant stated:

I loved it [the text messaging] because it kept me…accountable…because it kept asking the questions over and over again. In the beginning, it was like a little stressing because I was like, “Oh, my God, these messages, I don’t want to deal with it.” But, it kept me accountable. And it was good.

[Participant N36]

Similarly, another participant remarked:

I had to answer for all the cigarettes I smoked today and hold myself accountable. I thought that was ingenious.

[Participant N06]

In some instances (n=6), the text messages served as deterrents to impulsive smoking. One participant shared:

[There] were a couple of times when they [cigarettes] were right on hand…I’m upstairs, I’m getting ready to light and then boom [*sic*], oh, “did you smoke today? How many times did you smoke since we last communicated?”

[Participant N31]

Similarly, the anticipation of upcoming EMA messages served as a motivator for refraining from smoking or delaying the next cigarette. The participant went on to describe their morning routine:

…while you’re trying to wake up and organize yourself, you pick up the cigarette…[but] you know you’re going to get a text at 9 o’clock saying, “did you smoke?” right? So that notion pops in your head, so you don’t smoke right away.

[Participant N31]

Having the knowledge that there was a regular time for reporting their smoking activities encouraged participants to make more deliberate choices regarding their smoking behaviors. One participant discussed how they used EMA check-ins to consciously reduce their cigarette consumption during the time leading up to the EMA prompt:

I knew at a certain time, I was going to get this text. So, when I went to the text, I wanted to have everything in line. I wanted it to be right. So therefore, I would only smoke four cigarettes because that was the allotted cigarettes that I was supposed to smoke at the time. During that time, I would only smoke three cigarettes because I had cut down to three during that period of time and I looked forward to doing it.

[Participant N04]

### EMA Text Messages Can Negatively Impact Participants’ Emotions and Smoking Behaviors

#### Overview of Negative Impacts

A second major theme that emerged from the data was the potential negative effects that EMA texting could have on participants. Although many participants reported experiencing positive effects of EMA on their smoking behaviors, some participants (n=7) reflected on the potential negative impacts of the EMA text messages. This theme—the negative effects of EMA—was further distilled into the following two subthemes: the potential roles of EMA in (1) producing negative emotions and (2) triggering cigarette cravings.

#### Repeated Text Messages Asking About Smoking Behaviors Produce Negative Emotions

Some participants (n=4) mentioned feeling “bad” when they had to admit to smoking or experiencing relapses via the text messages. However, some respondents admitted that the guilt experienced as a result of the text messages, while being a negative emotion, reinforced their determination and prompted them to make renewed efforts, with one participant stating that the text messages “helped me because when I wrote how…I smoked a cigarette…I kind of felt bad” (Participant N58). Moreover, other participants emphasized the negative emotions and guilt experienced when a spotlight was focused on their perceived failures. One participant described the experience by saying, “being able to tell about my progress, or lack thereof, I would have felt bad if I had a slip up” (Participant N21).

#### Text Messages Inquiring About Cigarette Use May Have a Triggering Effect

Several participants (n=5) expressed their desire to remove the texting component of the program due to its triggering effect on their smoking. Some participants reported that the text messages acted as triggers for cigarette use, as the text messages reminded them of smoking, thereby eliciting an urge to smoke. One participant said that the EMA “was a reminder, actually,” and “…with the reminder came in the struggles” (Participant N20). Notably, another participant said:

…[the text messages] happened so often, and you knew they were coming, and they started to almost act like a trigger because you sometimes weren’t even thinking about [smoking], but then they would ask you about cigarettes and suddenly you’re thinking about it…they were more triggers to smoke than to prevent smoking.

[Participant N31]

## Discussion

### Principal Findings

This study indicates that smoking intervention participants perceive the collection of EMA smoking behavior data via SMS text messaging as a potential influence on their smoking behaviors. The themes developed from the analysis revealed that EMA, in the form of an SMS text messaging smoking diary, may be perceived as a source of accountability for smoking reduction but may also be a trigger for cigarette use among some people. The results of this study emphasize the need to examine the potential influence of EMA data collection techniques on participants’ behaviors within smoking interventions, as well as in other behavioral research.

The perceived impacts of EMA on smoking behaviors are consistent with previously made observations that the act of receiving EMA prompts can increase behavioral awareness and act as a trigger for craving [6-8], in addition to altering participants’ moods [2,12]. This suggests that EMA for data collection purposes has the potential to unintentionally create an assessment and intervention effects in itself. Although a lack of EMA impact on behavior has been reported in some studies [11,14,15], these studies may be limited by the choice of measures used. As seen in suicide research, EMA prompts have been observed to have an effect on some measures, such as mood, but a minimal effect on other measures, such as suicidal ideation [2]. Therefore, when designing a study, it is important for researchers to reflect on the various factors that may influence the behaviors of interest and consider the potential effect that EMA may have on these factors, in addition to the primary outcomes of interest. Due to its potential effect on participants, investigators should consider and discuss the potential for a behavioral influence to be introduced into a study through the use of EMA data collection.

There is a need for further investigation into the ways that and the degree to which EMA affects participants. Within the EMA literature, there is a general lack of discussion around the effects of EMA on participants’ behaviors. When seeking to improve EMA methods, focus is often placed on participant retention and the validity of the data collection method [20-23], with little to no discussion on the potential behavioral impact of EMA. Indeed, when discussing strengths and limitations of EMA, a large portion of the EMA study literature discusses and reports measures of adherence to and reliability of EMA data collection [20-23], with few studies exploring the potential limitation of EMA in which the data collection itself may affect behaviors of interest [2,5-8,11,12,14,15]. A potential effect from EMA may influence the interpretation of the results; therefore, investigators ought to be encouraged to report considerations related to EMA when designing and publishing a study. Future research may benefit from randomizing a subset of participants to receive one EMA modality (eg, texting) while observing behaviors among all participants with another measurement modality (eg, Bluetooth e-cigarette monitor).

When the potential for EMA to influence study outcomes is identified, less obtrusive EMA methods could be considered, when available. The participants in this study expressed the omnipresent awareness and anticipation of the SMS text messaging–based EMA. This awareness altered participants’ behaviors and resulted in negative emotions that likely would not have been emphasized had the EMA not been used or had been subtler. There are numerous types of EMA strategies used in smoking research [4]. Future intervention research studies could consider less frequent SMS text messaging or EMA data collection methods outside of SMS text messaging that may have a more minor impact on smoking behaviors, such as the use of biosensors [24], Bluetooth-enabled devices [25], or puff counters [26].

This study had a few limitations. First, the interview guide was not designed to investigate the impact of EMA on participants’ smoking behaviors. Therefore, further details on the effects of EMA were not deeply explored, limiting the scope of this analysis. The unprompted nature of the participants’ observations of behavioral impact, however, strengthens the conclusion that the SMS text messaging–based EMA had a meaningful impact on the trial participants. Second, the sample of interview participants was not randomized, and the interview was not required; rather, it was offered to all participants sequentially as an optional component. This potentially introduced selection bias, as those with stronger opinions on the program may have been more likely to participate. Third, as EMA data were collected as part of a smoking reduction trial, it is difficult to completely disentangle the effects of the intervention on changes in behavior from the effects of EMA. Finally, the impact of EMA on behavior change was based on the self-reported perceptions of interview participants, and behavior changes were not directly observed. Therefore, this study cannot be used as conclusive evidence that the EMA had a significant impact on smoking behaviors, and further research is needed.

### Conclusion

The collection of EMA smoking behavior data via SMS text messaging may influence the behaviors and perceptions of participants in smoking interventions. More research is needed to determine the magnitude of impact and mechanisms, to account for the potential effects of EMA on behavior change. Furthermore, a broader discussion of the behavioral influence introduced by the use of EMA may be warranted among the EMA research community.

## Supplementary material

10.2196/52122 Multimedia Appendix 1 Study in-depth interview guide.

10.2196/52122 Checklist 1 COREQ (Consolidated Criteria for Reporting Qualitative Research) checklist.

## Abbreviations

COREQ: Consolidated Criteria for Reporting Qualitative Research
EMA: ecological momentary assessment
NRT: nicotine replacement therapy
RCT: randomized controlled trial
