# Supplementary material for: Trial Participants’ Perceptions of the Impact of Ecological Momentary Assessment on Smoking Behaviors: Qualitative Analysis
Source: JMIR Mhealth Uhealth. 2024 Jan 16;12:e52122. doi: 10.2196/52122 (PMC10837109; doi:10.2196/52122)
Supplement: Multimedia Appendix 1 [file mhealth-v12-e52122-s001.docx]

EC COPD Study In-depth Interview Guide

**Before interview call, make sure an audio consent has been signed.**

**READ BEFORE RECORDING:**

“Thank you for taking the time to speak with me about your experience with our E-cigarette harm reduction program. We feel it is important to understand and hear about your experience. When I ask about the program today, I am referring to both the texting and the counseling sessions unless I specifically say otherwise, does this make sense? “

Wait for answer ……

**Great, with your permission I would like to audio record the interview today so I may listen back to it, is it ok that I start the recorder now?**

Wait for answer ……

**START RECORDING ON TWO DEVICES.**

“Thanks Mr/Mrs………… (**Last name**- you may be more personable after the end of the interview) for agreeing to take part in this interview, I just started the recording. There is no right or wrong answer, we only want to understand your experience to learn from it so that we can improve or modify the program moving forward. All information that you provide will be confidential and will be beneficial.”

**1- Participation (don’t read)**

- What was your main reason for deciding to take part in the study?
  - Probe: What were your hopes for taking part in the study?

**2- Program (don’t read)**

- What do you think about the program overall now that you have experienced it for a while?
  - Probe: What have been the most helpful aspects of participation? How did it help you?
  - Probe: What have been the least helpful aspects of participation?
  - Probe: What about something to try to improve? For those who do NOT want to say something negative or critical >> If we were to do the program all over again, what would you do differently? What we could improve?
- **When was your last counseling session?** What happened in that appointment?
  - Probe: Have you had trouble scheduling any sessions?
  - What about the frequency of sessions?
  - How do you find the length of time of the counseling sessions? Sufficient?
  - What do you think conducting it over video? Prefer phone or video? What is the difference?
  - How do you feel about the experience of the counselor that was providing session? Is she helpful? Anything that she could have done differently?
  - What do you think about Video counseling? What about it compared to Phone? Any difficulties? What did you like about it?
- **During the program, you answered questions over text on daily basis-** What was that experience like for you?
  - Probe: What do you think about the text messaging that was used in the study?
  - Frequency?
  - Your thoughts about it?
  - How may texting have affected your overall experience with the program?

**3- Acceptability (don’t read)**

- What aspects do you like the best about the program?
  - What aspects do you like the least?
- Are you satisfied with the results since you participated?
  - Has this program met your expectations? **Tell me more?**
- Are there things you would change about how it is delivered or experienced?
  - If so, what?
  - What about the text messages specifically?
- **Would you recommend this program to a friend?**
  - Why or why not? (generalizability- make sure you ask this question even if the patient is literally saying we were the best – so that we can report the count in a paper)

**4- Impact (don’t read)**

- Has your lifestyle changed since you started the program? If so how?
- How do you feel about your smoking pattern now?
  - How much do you think your cigarette use changed over the past few weeks? When was the last time you smoked a cigarette?
  - How many cigarettes you smoke now? And how likely this may change in the future?
  - If so, in what ways? Have you change the number of cigarettes you use since you started?
  - Have you noticed any **positive effects** from e-cigarette/NRT use? (Probe: positive changes you may have noticed- less coughing, improved breathing, better physical fitness?)
  - How about any **negative effects** from e-cigarette/NRT use? Negative changes you may have noticed- more coughing, worse physical fitness, headache, dizziness.
- How do you believe the use of e-cigarettes/NRT has helped you reduce your cigarette intake? Why you think this happened?
- How likely will you continue using e-cigarettes/NRT in the future? And why?
  - Are you planning to continue using e-cigarettes after the program stops? Why?
  - Will you try to switch to e-cigarette completely?
  - Try different e-cigarettes?

**5-USER EXPERIENCE (don’t read)**

- Overall, what do you like about e-cigarettes/NRT?
  - What **maybe not so good** about using e-cigarettes/NRT?
  - Technical problem with E-cigarettes?
- What are your thoughts about **E-cigarettes/NRT** in comparison to cigarettes?
- **How does your experience with e-cigarette/NRT compare to your prior experiences to cutting back or trying to quit cigarette smoking in the past?**
- How do you think the program may have changed your cigarette use?
  - Do you think e-cigarettes/NRT is a good way to quit or cut back on smoking? And why do you think so?

**ALWAYS ASK THE FOLLOWING:**

Thanks so much for your time so far, this information will be very helpful, I have two more questions.

1. Is there anything else you would like to tell us about the experience of the overall therapy program that we haven’t covered?
2. Considering all our discussion, how could our counseling and texting program be improved from your perspective?

THANKS a lot for your time, **I will now stop the recording.**

Now say thanks again, and confirm that there will be a final follow up in few weeks as part of the study.
