# Supplementary material for: Trial Participants’ Perceptions of the Impact of Ecological Momentary Assessment on Smoking Behaviors: Qualitative Analysis
Source: JMIR Mhealth Uhealth. 2024 Jan 16;12:e52122. doi: 10.2196/52122 (PMC10837109; doi:10.2196/52122)
Supplement: Checklist 1 [file mhealth-v12-e52122-s002.docx]

Consolidated criteria for reporting qualitative studies (COREQ): 32-item checklist

| **No** | | | **Item** | | | | | **Guide questions/description** | | | | **Page** |
| --- | --- | --- | --- | --- | --- | --- | --- | --- | --- | --- | --- | --- |
| **Domain 1: Research team and reflexivity** | | | | | | | | | |  |  |  |
| Personal Characteristics | | | | |  | | |  | | | |  |
| 1. | Interviewer/facilitator | | | | | | | Which author/s conducted the interview or focus group? | | | | 7 |
| 2. | Credentials | | | | | | | What were the researcher's credentials? *E.g. PhD, MD* | | | | title |
| 3. | Occupation | | | | | | | What was their occupation at the time of the study? | | | | 7 |
| 4. | Gender | | | | | | | Was the researcher male or female? | | | | n/a |
| 5. | Experience and training | | | | | | | What experience or training did the researcher have? | | | | n/a |
| Relationship with participants | | | | | |  | |  | | | |  |
| 6. | | Relationship established | | | | | | Was a relationship established prior to study commencement? | | | | n/a |
| 7. | | Participant knowledge of the interviewer | | | | | | What did the participants know about the researcher? e*.g. personal goals, reasons for doing the research* | | | | n/a |
| 8. | | Interviewer characteristics | | | | | | What characteristics were reported about the interviewer/facilitator? e.g. *Bias, assumptions, reasons and interests in the research topic* | | | | n/a |
| **Domain 2: study design** | | |  | | | | |  | | | |  |
| Theoretical framework | | | |  | | | |  | | | |  |
| 9. | | | Methodological orientation and Theory | | | | | What methodological orientation was stated to underpin the study? *e.g. grounded theory, discourse analysis, ethnography, phenomenology, content analysis* | | | | 7-8 |
| Participant selection | | | |  | | | |  | | | |  |
| 10. | | | Sampling | | | | | How were participants selected? *e.g. purposive, convenience, consecutive, snowball* | | | | 6 |
| 11. | | | Method of approach | | | | | How were participants approached? e*.g. face-to-face, telephone, mail, email* | | | | 6 |
| 12. | | | Sample size | | | | | How many participants were in the study? | | | | 7 |
| 13. | | | Non-participation | | | | | How many people refused to participate or dropped out? Reasons? | | | | n/a |
| Setting | | |  | | | | |  | | | |  |
| 14. | | | Setting of data collection | | | | | Where was the data collected? e*.g. home, clinic, workplace* | | | | 7 |
| 15. | | | Presence of non-participants | | | | | Was anyone else present besides the participants and researchers? | | | | n/a |
| 16. | | | Description of sample | | | | | What are the important characteristics of the sample? *e.g. demographic data, date* | | | | 9 |
| Data collection | | |  | | | | |  | | | |  |
| 17. | | | Interview guide | | | | | Were questions, prompts, guides provided by the authors? Was it pilot tested? | | | | Supplement |
| 18. | | | Repeat interviews | | | | | Were repeat interviews carried out? If yes, how many? | | | | n/a |
| 19. | | | Audio/visual recording | | | | | Did the research use audio or visual recording to collect the data? | | | | 7 |
| 20. | | | Field notes | | | | | Were field notes made during and/or after the interview or focus group? | | | | n/a |
| 21. | | | Duration | | | | | What was the duration of the interviews or focus group? | | | | 6 |
| 22. | | | Data saturation | | | | | Was data saturation discussed? | | | | 6 |
| 23. | | | Transcripts returned | | | | | Were transcripts returned to participants for comment and/or correction? | | | | n/a |
| **Domain 3: analysis and findings** | | | | | | |  | |  | | |  |
| Data analysis | | |  | | | | |  | | | |  |
| 24. | | | Number of data coders | | | | | How many data coders coded the data? | | | | 8 |
| 25. | | | Description of the coding tree | | | | | Did authors provide a description of the coding tree? | | | | n/a |
| 26. | | | Derivation of themes | | | | | Were themes identified in advance or derived from the data? | | | | 8 |
| 27. | | | Software | | | | | What software, if applicable, was used to manage the data? | | | | 7 |
| 28. | | | Participant checking | | | | | Did participants provide feedback on the findings? | | | | n/a |
| Reporting | | |  | | | | |  | | | |  |
| 29. | | | Quotations presented | | | | | Were participant quotations presented to illustrate the themes / findings? Was each quotation identified? e*.g. participant number* | | | | 9-13 |
